# A lightweight deep-learning model for parasite egg detection in microscopy images

**DOI:** 10.1186/s13071-024-06503-2

**Published:** 2024-11-06

**Authors:** Wenbin Xu, Qiang Zhai, Jizhong Liu, Xingyu Xu, Jing Hua

**Affiliations:** 1 Nanchang Key Laboratory of Medical and Technology Research, Nanchang University, Nanchang, China; 2 School of Mechanical and Energy Engineering, Zhejiang University of Science and Technology, Hangzhou, China; 3 Shaoxing Institute of Advanced Research, Wuhan University of Technology, Shaoxing, China; 4 School of Software, Jiangxi Agricultural University, Nanchang, China

**Keywords:** Parasite eggs, Deep learning, Object detection, AFPN, Lightweight design

## Abstract

**Background:**

Intestinal parasitic infections are still a serious public health problem in developing countries, and the diagnosis of parasitic infections requires the first step of parasite/egg detection of samples. Automated detection can eliminate the dependence on professionals, but the current detection algorithms require large computational resources, which increases the lower limit of automated detection. Therefore, we have designed a lightweight deep-learning model, YAC-Net, to achieve rapid and accurate detection of parasitic eggs and reduce the cost of automation.

**Methods:**

This paper uses the ICIP 2022 Challenge dataset for experiments, and the experiments are conducted using fivefold cross-validation. The YOLOv5n model is used as the baseline model, and then two improvements are made to the baseline model based on the specificity of the egg data. First, the neck of the YOLOv5n is modified to from a feature pyramid network (FPN) to an asymptotic feature pyramid network (AFPN) structure. Different from the FPN structure, which mainly integrates semantic feature information at adjacent levels, the hierarchical and asymptotic aggregation structure of AFPN can fully fuse the spatial contextual information of egg images, and its adaptive spatial feature fusion mode can help the model select beneficial feature and ignore redundant information, thereby reducing computational complexity and improving detection performance. Second, the C3 module of the backbone of the YOLOv5n is modified to a C2f module, which can enrich gradient information, improving the feature extraction capability of the backbone. Moreover, ablation studies are designed by us to verify the effectiveness of the AFPN and C2f modules in the process of model lightweighting.

**Results:**

The experimental results show that compared with YOLOv5n, YAC-Net improves precision by 1.1%, recall by 2.8%, the F1 score by 0.0195, and mAP_0.5 by 0.0271 and reduces the parameters by one-fifth. Compared with some state-of-the-art detection methods, YAC-Net achieves the best performance in precision, F1 score, mAP_0.5, and parameters. The precision, recall, F1 score, mAP_0.5, and parameters of our method on the test set are 97.8%, 97.7%, 0.9773, 0.9913, and 1,924,302, respectively.

**Conclusions:**

Compared with the baseline model, YAC-Net optimizes the model structure and simplifies the parameters while ensuring the detection performance. It helps to reduce the equipment requirements for performing automated detection and can be used to realize the automatic detection of parasite eggs under microscope images.

**Graphical Abstract:**

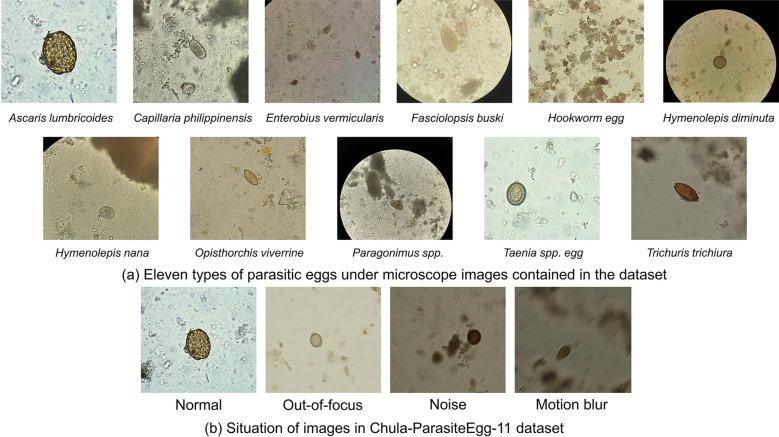

## Background

Intestinal parasitic infections (IPIs) caused by protozoa and helminth parasites are one of the most common infectious diseases, and soil-transmitted helminth (STH) infection is the main cause of IPIs [1, 2]. IPIs are still prevalent in countries with poor sanitation conditions due to shortage of sanitation facilities, poor sanitation conditions, and neglect of health education [3, 4]. Children, pregnant women, and patients with low immune function are high-risk groups for infection with IPIs. According to the World Health Organization’s 2023 statistics, there are currently approximately 1.5 billion STH-infected individuals worldwide, with over 900 million children and 138.8 million pregnant and lactating women living in areas with soil-transmitted helminths, requiring treatment, prevention, and intervention [1, 5]. If patients with IPIs are not treated promptly, they may lead to vomiting, diarrhea, weakness, impaired child development, adverse pregnancy outcomes, etc., which seriously affect people’s daily lives and will harm the country’s economy, fertility, and development in the long run [3, 6]. Therefore, timely detection and precise intervention of IPIs have positive effects at both the individual and social levels.

Microscopic parasitic examination is the gold standard for parasitic diagnosis [7]. It is usually performed by experienced laboratory physicians who examine sample smears under a microscope to identify the type of parasites and eggs present, which serves as the basis for diagnosing parasitic diseases. Manual microscopic examination has problems such as low efficiency, high workload, odor leakage, poor working environment, and the accuracy of the test results is closely related to the prior knowledge and physical condition of the doctor [8]. The automation of the detection process can not only provide accurate and fast results but also provide identification services for a wider range of people who lack professional knowledge. Considering that different parasites and eggs have obvious species characteristics and good morphological performance under the microscope [9], the use of digital image processing models based on artificial intelligence to automatically identify parasites and eggs in microscope images is currently a hot development direction for improving the current status of parasite detection.

The automated detection of parasites and eggs is the result of years of deep integration of medical inspection technology and image processing technology. The development process of automated detection can be divided into two stages according to the methods used. The first stage is the semiautomatic detection stage based on traditional machine learning; the other stage is the automatic detection stage based on convolutional neural network (CNN).

### Traditional machine learning in parasite egg detection

In the early stage of the development of image recognition technology, the detection of parasite eggs mainly relied on morphology and threshold segmentation methods for rough localization of the eggs and then further distinguished the type of eggs by linear analysis of the image features of the located area [10]. Early detection methods had numerous steps and poor performance. With the rise of machine learning (ML), ML models that self-optimize and gradually approach the optimal solution during the iteration process have been deeply applied to image processing tasks, which has significantly improved the accuracy of parasite egg image detection results based on feature analysis, and the robustness and generalization ability of machine learning models are also much stronger than traditional analysis methods. However, the parasite egg analysis method based on traditional machine learning models still cannot avoid the need to locate the eggs specifically and manually extract features from the egg pixel area during preprocessing [11, 12]. Effective feature information is the prerequisite for the high performance of machine learning methods, but the type and quality of features manually designed and extracted depend largely on the operator’s professional knowledge, and manual operation cannot be truly objective and comprehensive, so it is impossible to fully utilize the valuable information in the image [13, 14]. Therefore, the egg detection technology based on traditional machine learning models relies too much on human intervention and cannot truly realize the automated and intelligent detection of eggs.

### Convolutional neural network in parasite egg detection

The rapid development of deep learning has once again pushed artificial intelligence to the forefront of science. As a branch of deep learning, CNN is different from traditional machine learning methods. It can directly extract feature information from input images and directly output analysis results, fundamentally avoiding the bias caused by subjective feature selection on the algorithm. Therefore, applying it to parasite egg detection tasks can achieve end-to-end automated detection. Banerjee et al. [15] proposed a deep CNN for detecting malarial parasites in blood smears. AlDahoul et al. [16] combined CNN with an attention module to classify parasite eggs. Huo et al. [17] identified parasite eggs in microscope images based on the YOLOv5 [18] model. Kumar et al. [19] used different CNN models combined with image processing methods to classify parasites. Their experimental results verified that CNN can achieve accurate detection of parasites and eggs. From previous studies, it can be concluded that CNN has been used as a good choice for parasite and egg detection research because of its powerful feature learning ability and end-to-end task processing mode.

Object detection algorithms can be divided into two-stage detectors and one-stage detectors according to the detection process [20]. The most representative two-stage detector is the R-CNN series algorithm, while the representative one-stage detector is the YOLO series detection algorithm. Currently, the egg detection algorithm is also mainly developed around these two algorithms. Viet et al. [21] identified eggs from microscope images based on Faster-RCNN. They used Faster-RCNN to detect images directly and only fine-tuned the learning rate and the number and size of anchors, which was not novel enough. Ruiz-Santaquiteria et al. [22] integrated multiple detection algorithms such as Faster-RCNN, Cascade M-RCNN, and YOLOX into a deep-learning model for egg recognition. Although their aggregated network achieved the best performance, the improvement was limited compared with a single model, and the model structure was very complex and required high computing resources to support it. Pedraza et al. [23] used ResNet-50 or Swin-Transformer [24] as new feature extractor based on Mask RCNN and Cascade Mask RCNN and conducted comparative experiments to obtain the model structure with the best performance in egg detection. Using appropriate feature extractors can indeed improve model performance, but their experiments only considered two types of feature extractors and had limited experimental content.

The RCNN series algorithms are widely recognized to have higher detection performance than YOLO, but their complex structure and high computational requirements cannot be ignored. Now, the most urgent need for egg detection is in remote and poor areas with poor sanitary conditions, so from the perspective of popularization, the YOLO-based model with lower hardware requirements is the first choice for parasite and egg detection. Abdurahman et al. [25] used modified YOLOv3 and YOLOv4 models for malaria parasite detection in microscopy images of blood smears and compared the detection results with Faster RCNN and SSD. The only modification they made to the model was to change the size of the detection head, and they used an intersection of union threshold of 0.3 in the experiment, which resulted in artificially high detection results. Kumar et al. [26] used YOLOv5 to detect and classify intestinal parasite eggs. However, they used early CNNs such as SSD and AlexNet to compare with YOLOv5, resulting in insufficient credibility of the results. Wan et al. [27] designed a Coupled Composite Backbone Network (C2BNet) of a one-stage detector for worm egg recognition. The backbone of C2BNet is formed by coupling and compositing 8 Swin-Transformer blocks and 8 CNN blocks, which can provide a powerful feature extraction capability for the model and significantly improve the model detection performance. Inevitably, the number of parameters and computational complexity of the model are very large.

For intelligent and automated parasite egg detection, computing power cost is only part of the detection cost. The hardware required for automated image acquisition, such as microscopes, *X*–*Y* axis mobile platforms, and high-definition cameras or integrated paddle scanners, is the bulk of the cost. However, we do not have the ability to produce and process hardware. Therefore, in terms of software, we are committed to ensuring the detection performance of the model under the conditions of low computing power and low image resolution, which comprehensively reduces the hardware requirements for automated detection of parasite eggs and promotes the popularization of detection in remote and impoverished areas, enabling local people to detect diseases earlier and receive timely treatment.

At the current stage of work, this paper aims to provide a method for automatic detection of parasite eggs under microscope images with low computing power, which also having a certain detection capability for low-resolution and blurred egg images. This can lay a solid foundation for future implementation of egg image detection under low resolution and low computing resources. Our main contributions in this work are summarized below:

1. We made lightweight improvements to the YOLOv5 model using asymptotic feature pyramid network (AFPN) and C2f modules and ultimately designed a CNN network called YAC-Net for parasite egg detection. The former can asymptotically and adaptively fuse multilevel features, making the spatial contextual information of egg images more effectively utilized in the model. The latter improves the feature extraction ability of the model by enriching the gradient information.
2. We performed a comparative experiment on egg detection, comparing the performance of the designed YAC-Net with the state-of-the-art algorithms in the YOLO series. The experimental results confirm that compared with lightweight detection methods of the same level, YAC-Net’s detection performance is at the forefront, and it can also perform good detection on blurred and out-of-focus images.
3. We experimentally explored the effect of using only the AFPN module or the C2f module on the model detection performance, and the paired *t*-test results confirmed that the performance improvement of YAC-Net compared with the baseline model is statistically significant.

The rest of the article is arranged as follows. The “Methods” section introduces the designed convolutional neural network structure. The “Dataset and experimental setup” section provides the data source, data augmentation method, experimental environment, and parameter settings of the experiment. The “Results” section shows the experimental process and prediction results of the egg detection experiment and compares the performance with other methods. In the “Discussion section”, we discuss our findings and ideas in the experiment. The final “Conclusions” section summarizes the paper and explains our future research directions.

## Methods

### Overview of model architecture

**Fig. 1:**
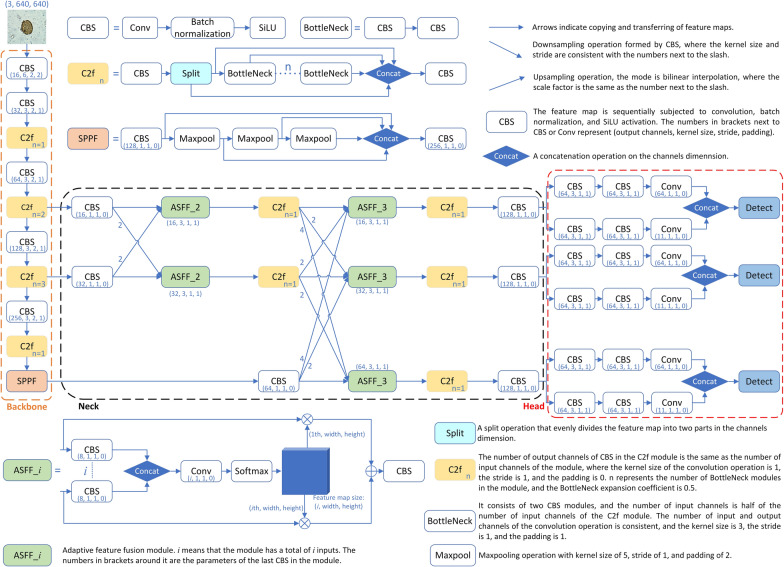
Overview of YAC-Net’s model structure. The model is mainly composed of the backbone, neck, and head. The backbone is mainly used to extract features from the input image. The neck further processes the feature information extracted by the backbone and, finally, provides it to the head to achieve specific object detection

The YOLOv5n + AFPN + C2f network model we designed, hereinafter referred to as YAC-Net, can be divided into three parts according to the structural function: backbone, neck, and head. As an important component of the object detection network, the backbone mainly extracts the features of image. Considering that in the detection task, it is also necessary to identify the categories of objects in the image. Therefore, in some research works, researchers will directly use the image classification network as the backbone of the model [23]. The function of the neck in the model is to fuse the deep semantic feature information extracted by the backbone with the shallow image detail information to improve the detection performance of the model. The AFPN [28] has the characteristics of multiscale feature fusion, which is very beneficial to the feature learning of the model. Therefore, we build the AFPN structure on the basis of the backbone to form the neck part of the model. The head makes the final prediction result based on the feature information fused by the neck network. Figure 1 shows the overall architecture of the proposed deep-learning model for parasite egg detection in microscopy images.

### Network architecture

As an object detection network model, YAC-Net is mainly composed of convolutional layers that are combined, sorted, and stacked. By combining elements such as pooling layers and upsampling layers, the feature information in the images fed to the model can be accurately captured, fused, and learned step by step. Finally, end-to-end object detection is achieved from input to output.

The convolutional layer mainly consists of convolution kernels (filters), batch normalization, and non-linear activation functions. The function of filters is to extract the feature information of the egg image. As the number of convolutional layers increases, the feature information extracted by the filter will change from the shallow semantic feature information to the deep edge feature information. The more filters there are, the more information is extracted but the more computing resources are required, and it is also more likely to cause model overfitting. The goal of batch normalization is to normalize the feature map so that the input of each layer keeps the same distribution as much as possible and falls into the gradient-sensitive area of the activation function, which can avoid the problem of gradient vanishing and make the model converge faster. The role of non-linear activation function in neural network is mainly to introduce non-linear characteristics so that the network can approximate any non-linear function. The activation function used in our model is the Sigmoid linear unit (SiLU), which is used in the convolution layer to perform non-linear mapping transformation on the feature map formed after convolution operation and batch normalization.

The pooling layer mainly downsamples the feature map, which can not only reduce the amount of data and computational complexity but also retain important feature information. The proposed model architecture uses the maxpooling operation to sample the feature map.

The model learns multiscale feature information, which can improve the performance of detection and the ability to capture image details. To aggregate feature maps of different scales, it is necessary to use upsampling operations to upsample low resolution feature maps to high resolution. In this experiment, bilinear interpolation is used to upsample feature maps.

#### Neck with AFPN structure

**Fig. 2:**
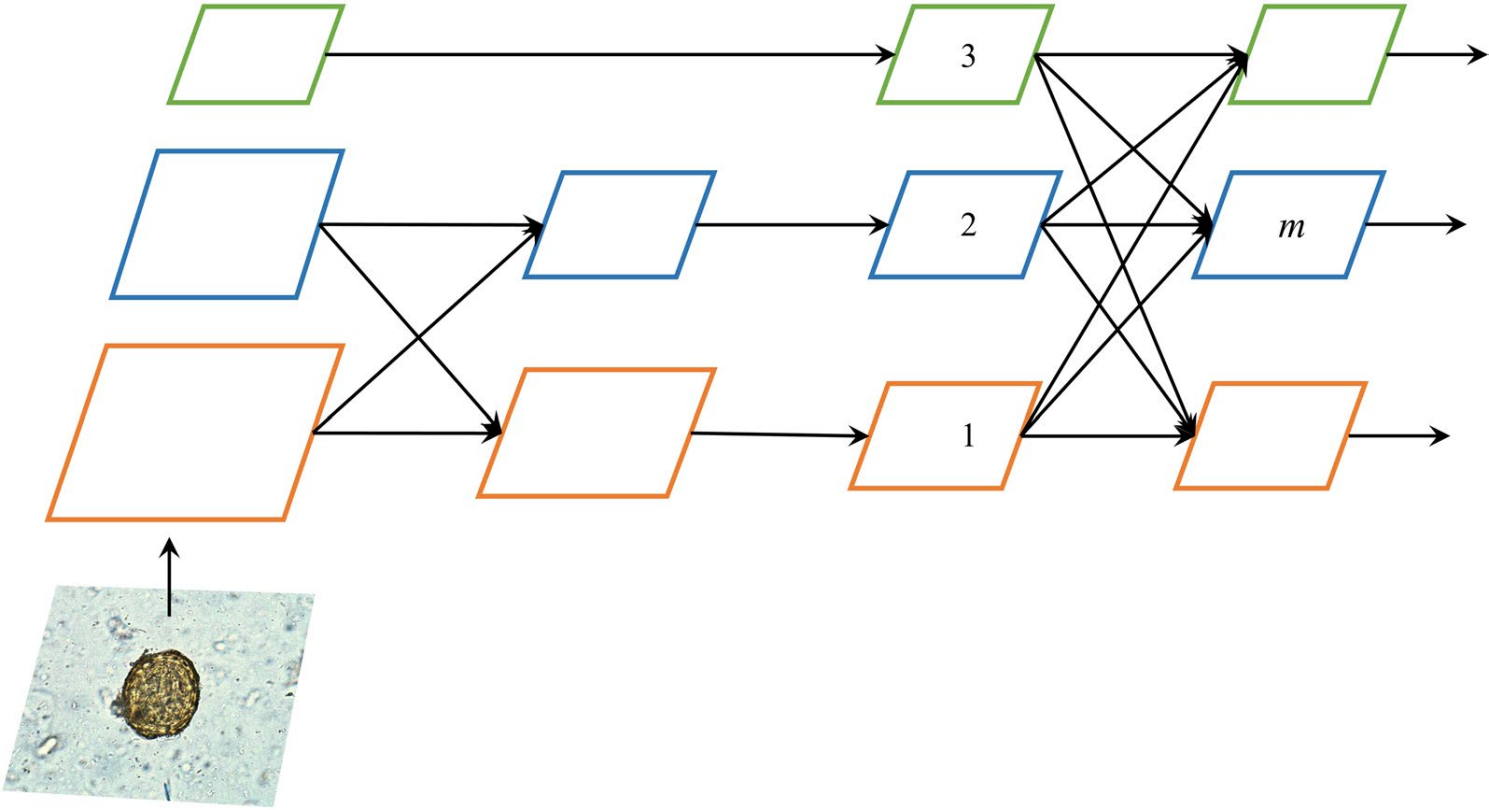
A structural sketch of the AFPN in the neck of the proposed model. The specific implementation of AFPN in the proposed model is shown in the neck of Fig. 1

YOLOv5’s neck uses the structure of feature pyramid network (FPN) and path aggregation network (PAN). The FPN structure can only fuse features of adjacent layers and cannot fuse feature information with a large span. The PAN structure fuses spatial context information from the bottom up, which may cause feature information loss or degradation and does not fully utilize the information extracted by the backbone. In the bottom-up feature extraction process, AFPN combines two low-level features of different resolutions for fusion in the first stage; then in the later stage, high-level features are gradually added to the fusion process, and the top-level features are fused at the end. This mode avoids the large information gap between nonadjacent feature layers. The bottom-level features are fused with the high-level features for semantic information, and the high-level features are fused with the bottom-level features for detail information. This structure can prevent the loss of feature information during multilevel transmission. During the entire feature fusion process, there may be contradictory object information between different feature layers. AFPN uses adaptive spatial fusion operations to solve this problem. AFPN assigns different spatial weights to features at different levels during the asymptotic feature fusion process, highlighting the importance of key levels and reducing the impact of contradictory information of different objects. The AFPN structure built by the neck of the YAC-Net is shown in Fig. 2 below. The experiment integrates features from three levels. Assume that ${x}_{ij}^{n\to m}$ is the feature vector from the *n*-th layer to the *m*-th layer position (*i*, *j*), and the feature vector ${y}_{ij}^{m}$ obtained by the *m*-th layer is obtained through adaptive spatial fusion operation, which is a linear combination of the feature vectors ${x}_{ij}^{1\to m}$, ${x}_{ij}^{2\to m}$, ${x}_{ij}^{3\to m}$:

$${y}_{ij}^{m}={\alpha }_{ij}^{m}\cdot {x}_{ij}^{1\to m}+{\beta }_{ij}^{m}\cdot {x}_{ij}^{2\to m}+{\gamma }_{ij}^{m}\cdot {x}_{ij}^{3\to m},$$

${\alpha }_{ij}^{m}$, ${\beta }_{ij}^{m}$ and ${\gamma }_{ij}^{m}$, respectively, represent the spatial weights of features at each level in the *m*-th layer, satisfying ${\alpha }_{ij}^{m}$+${\beta }_{ij}^{m}$+${\gamma }_{ij}^{m}$=1.

#### C2f module structure

**Fig. 3:**
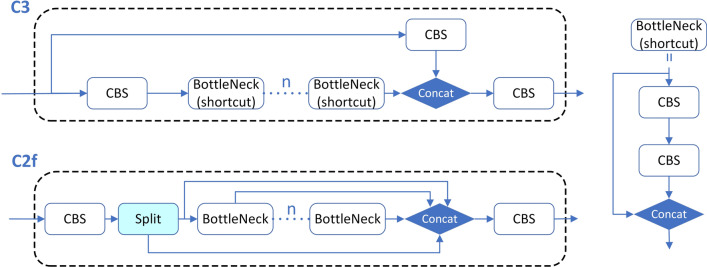
Structure diagram of C3 module and C2f module. The CBS, Concat, and other modules in the figure have the same meaning as in Fig. 1

The C3 module structure of YOLOv5n is composed of two branches and adopts a residual structure connection. It has a weak ability to process the feature details of small targets. The newly proposed C2f module refers to the structure of the C3 module and combines the idea of ELAN [29]: (1) by splitting the input feature map in the channel dimension, performing convolution operations on part of it and then concatenating them, this structure can effectively integrate features of different scales and retain more spatial and semantic information, and (2) the module structure takes into account the longest gradient path and the shortest gradient path, so that the model can obtain richer gradient flow information while ensuring lightweight. In fact, although C2f emphasizes keeping the model lightweight, it still has more parameters compared with C3. It is worth mentioning that in C2f, the expansion factor *e* can be used to adjust the number of channels in the intermediate hidden layer, which is an effective way to balance computational costs and model capacity. In our experiment, the C2f module will replace the C3 module in the model to improve the model’s ability to analyze the feature information of small targets. In addition, to improve the lightweight degree of the model, the expansion factor *e* in the C2f module and the BottleNeck block in it was set to 0.5. The C3 and C2f module structures are shown in Fig. 3.

## Dataset and experimental setup

### Data acquisition

**Fig. 4:**
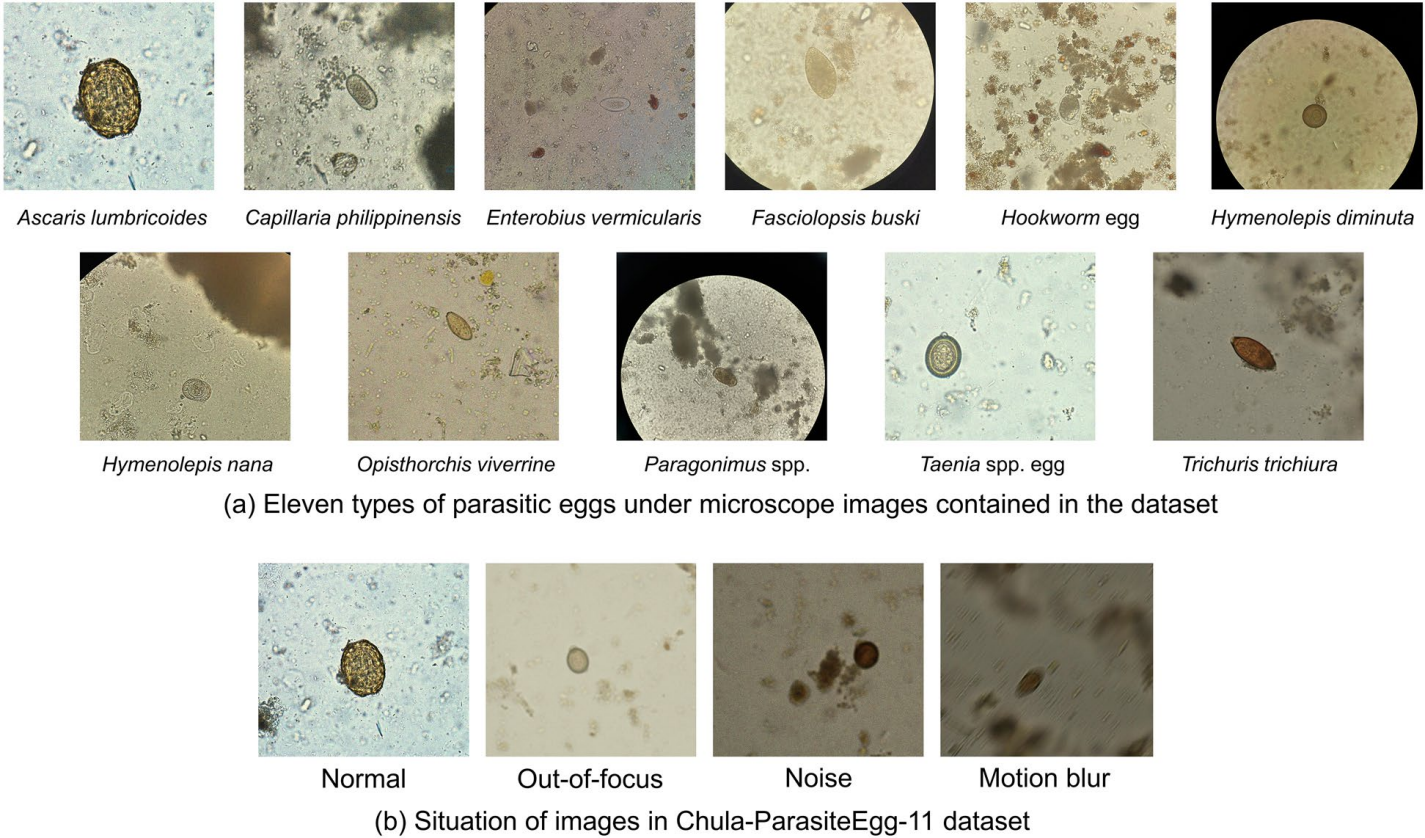
Parasite egg images selected from the Chula-ParasiteEgg-11 dataset. **a** The 11 parasite eggs contained in the dataset. **b** The presence of normal, out-of-focus, noise, and motion-blur images in the dataset

The experimental data for this experiment come from the ICIP 2022 Challenge: Parasitic Egg Detection and Classification in Microscopic Images [30]. The dataset contains 13,200 microscopic images of 11 species: *Ascaris lumbricoides*, *Capillaria philippinensis*, *Enterobius vermicularis*, *Fasciolopsis buski*, Hookworm egg, *Hymenolepis diminuta*, *Hymenolepis nana*, *Opisthorchis viverrine*, *Paragonimus* spp., *Taenia* spp. egg, and *Trichuris trichiura*. The average size of these eggs ranges from 15 to 140 μm. The eggs of various parasites are shown in Fig. 4a. These microscopic images were acquired by different devices, and the illumination and resolution of the images were different. Some images were out-of-focus, noisy, and had motion blur, as shown in Fig. 4b. These different conditions make the detection of eggs more difficult and closer to the actual detection environment, which helps to test the robustness of the model.

### Evaluation metrics

To evaluate the performance of the YAC-Net, the four metrics of Precision, Recall, F1 score, and mean average precision (mAP) were selected for evaluation on the test set. Precision indicates the ratio of the number of eggs correctly predicted as a certain category to the number of eggs predicted as that category. Recall indicates the ratio of the number of eggs correctly predicted in a certain category to the total number of eggs in that category. F1 score is the harmonic mean of precision and recall, which is a comprehensive evaluation metric that avoids a single maximum of precision or recall and is used to reflect the overall performance of the model. AP_θ represents the area under the P–R curve of a certain category predicted by the model when the intersection of union between the predicted box and the ground truth is greater than the threshold *θ*. It is used to measure the detection ability of the model on the category of interest. mAP_θ is the average AP of all detected categories and is a metric for comprehensive evaluation of model performance in the entire detection task. They are defined as:

$$\text{Precision}=\frac{\text{TP}}{\text{TP}+\text{FP}}\times 100\text{\%},$$

$$\text{Recall}=\frac{\text{TP}}{\text{TP}+\text{TN}}\times 100\text{\%},$$

$$\text{F}1\text{ score}=2\frac{\text{Precision}\times \text{Recall}}{\text{Precision}+\text{Recall}},$$

$$\text{mAP}=\frac{1}{n}\sum_{i=1}^{n}{AP}_{i},$$

where TP, FP, and TN are the number of true positive, false negative, and true negative results of the model for a certain type of parasite eggs, respectively. *n* is the number of all egg types, which is 11 in this paper.

### Experimental setup

This experimental environment is built using the Pytorch deep-learning framework. The experimental environment is Intel(R) Core(TM) i7-9750H, NVIDA GeForce RTX 2070 8G, 16G RAM, the operating system is Window10, the CUDA version is 10.0, the deep-learning framework is pytorch 1.10.0, and the Python version in the experimental environment is 3.8. To obtain the optimal model parameters, the model training process is 50 epochs, the initial learning rate is 0.01, the learning rate is adjusted using the cosine annealing method, the batch size is 8, the optimizer is SGD, and the momentum is set to 0.94. Finally, we save the weight parameters that obtain the highest value on mAP_0.5 on the validation set during the training process and use them for the subsequent test set to test the performance of parasite egg detection.

To ensure the rigor of the experimental results, the experiment was conducted using a fivefold cross-validation method. First, 20% of the data were extracted as the test set, then 10% of the data were randomly selected from the remaining 80% of the data as the validation set, and the remaining data were used as the training set. This dataset division method was performed five times in total, and the test set data were different each time to ensure that all data were used as a test set to participate in the model performance test. Finally, our experimental data consisted of 9504 images in the training set, 1056 images in the validation set, and 2640 images in the test set.

## Results

### Experimental result

**Fig. 5:**
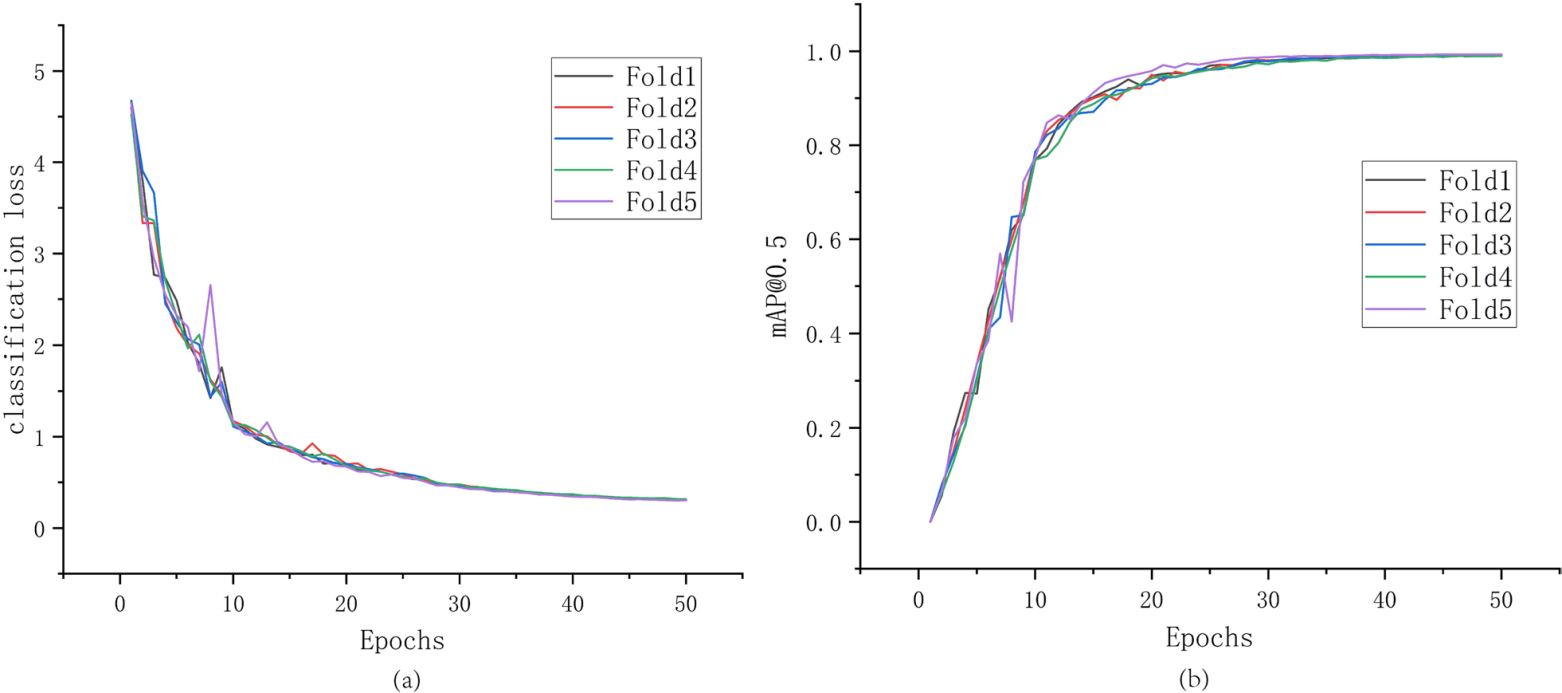
Performance curve of the model on the validation set during training process. **a** The classification loss function curve of the validation set. **b** The F1 score curve of the validation set

The performance curve of the proposed YAC-Net in the parasite egg detection experiment is shown in Fig. 5, and the detection results of the model in the test set are presented in Table 1. The data curves in Fig. 5 are all the performance of the model in the validation set during the training process. The validation set data do not participate in the model training but is only to intuitively reflect the performance changes of the model during training.

**Table 1:** The performance of YAC-Net on the test set under fivefold cross-validation

	Precision (%)	Recall (%)	F1 score	mAP_0.5
Fold1	98.0	97.4	0.9766	0.9906
Fold2	97.5	97.5	0.9747	0.9911
Fold3	98.0	97.7	0.9782	0.9911
Fold4	97.2	97.7	0.9746	0.9909
Fold5	98.4	98.0	0.9822	0.9926
Average	97.8	97.7	0.9773	0.9913

As can be seen from Fig. 5, as the number of iterations increases during training, the loss value is gradually decreasing, while mAP_0.5 is increasing. In the 0–25 epochs stage, the loss value and mAP_0.5 have obvious oscillations in the vertical direction, but the former shows an overall downward trend, and the latter shows an obvious upward trend. In 25–40 epochs, the vertical fluctuations of each curve have gradually stabilized, and the downward and upward trends have also become gentle. After 40 epochs, as the number of iterations increases, each curve has tended to be a straight line. The reason for this situation is the model’s weight parameters are constantly iterated, adjusted, and updated during the training process to reduce the gap between the model’s output and the ground truth. Since the iteration of parameters is based on a batch of data, and learning a batch of data may not always produce beneficial results, the model may go a long way. As the model learns more and more data, it will eventually get closer and closer to the ground truth. Therefore, this situation will show large oscillations in the loss curve, but the overall trend of the curve is still downward. As the training progresses, the learning ability of the model begins to saturate, and the parameters also oscillate around the optimal solution, making the loss value change smaller and smaller and finally fluctuating within a very small range. The above situation is reflected in the detection performance that the rising trend of mAP_0.5 curve changes from a significant oscillating rise to a small range of fluctuations and finally tends to a straight line. Finally, the highest values of mAP_0.5 in each fold of the model during the fivefold cross-validation training process are 0.9899, 0.9916, 0.9927, 0.9899, and 0.9932, respectively.

Table 1 presents the prediction results of the model on the test set after fivefold cross validation. We use precision, recall, F1 score, and mAP_0.5 to comprehensively evaluate the model’s detection performance for parasite eggs. In Table 1, the precision, recall, and F1 score of the model in each fold are all above 97%, and mAP_0.5 exceeds 0.99. Analysis of various metrics shows that YAC-Net can correctly capture the location and classify the 11 types of parasite eggs in microscope images with an accuracy of more than 97%, which can fully realize the automatic detection of parasite eggs. Combined with the optimal result of mAP_0.5 in Fig. 5, we can see that during the fivefold cross-validation process of the model, the final results of each fold show that the performance deviation of the model after five trainings is not large, which shows that the division of the dataset is not obviously biased and the robustness of the model is good. The mAP_0.5 of the model on the validation set is very similar to the result on the test set, and there are results on the test set that are higher than those on the validation set, which reflects that the model has outstanding generalization ability and can also detect and identify egg images that have not been touched.

### Comparison with state‑of‑the‑art methods

**Fig. 6:**
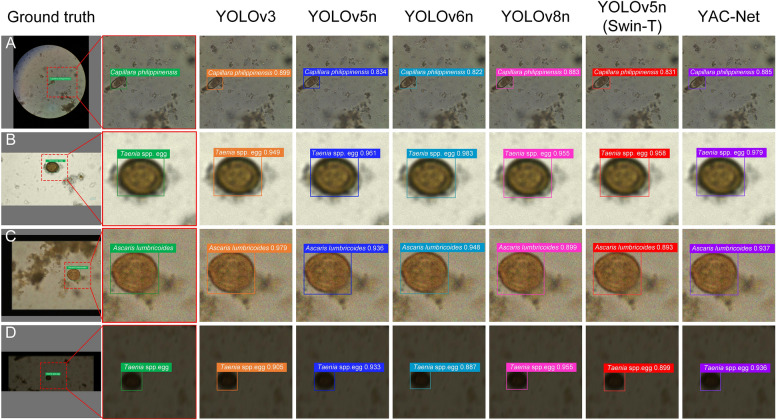
Visualization of detection of parasite egg images for different detection methods. **A**–**D** *Capillaria philippinensis* (**A**), *Taenia* spp. egg (**B**), *Ascaris lumbricoides* (**C**), and *Taenia* spp. egg (**D**), respectively. In addition, **A** is a normal image, **B** shows out-of-focus, **C** is a noisy image, and **D** is an image of parasitic eggs with motion blur. The number after the egg is the confidence score of the model in identifying eggs. In the case of accurate classification, the closer the number is to 1, the more accurate the recognition result is

To further verify the performance of the model, the YAC-Net algorithm is compared with several state‑of‑the‑art detection algorithms. YOLOv3 [31] adopts the design of prior frames and uses clustering algorithms to obtain multiple prior frames with different aspect ratios. It can achieve higher detection result but requires large computation. YOLOv6 [32] focuses on detection performance and reasoning efficiency. Its backbone adopts the idea of structural reparameterization to decouple the multibranch topological structure during training from the common structure during reasoning, achieving lower detection latency. YOLOv8 is one of the representative works of the new generation of excellent detection algorithms in the YOLO series. In addition, the Transformer model uses a self-attention mechanism for information interaction and performs well in image processing tasks. Many researchers have chosen Transformer as the backbone of object detection models [23, 27]. Here, we use the Tiny version of Swin-Transformer as the backbone of YOLOv5n, hereinafter referred to as YOLOv5n(Swin-T). Therefore, this experiment selected YOLOv3, YOLOv5n, YOLOv6n, YOLOv8n, and YOLOv5n(Swin-T) for control experiments on egg detection to further verify the performance of the YAC-Net algorithm. The experimental environment and data are consistent with those described in the “Dataset and experimental setup” section. The suffix *n* of the model indicates the smallest model in this series of algorithms. The experimental results are presented in Table 2, and the egg detection results of each algorithm are visualized in Fig. 6.

**Table 2:** YAC-Net model control experimental results

Model	Precision (%)	Recall (%)	F1 score	mAP_0.5	Parameters
YOLOv3	96.8	96.2	0.9651	0.9705	4,055,945
YOLOv5n	96.7	94.9	0.9578	0.9642	2,505,089
YOLOv6n	97.7	97.4	0.9765	0.9880	4,234,833
YOLOv8n	97.3	**98.0**	0.9754	0.9911	3,007,793
YOLOv5n(Swin-T)	96.9	95.7	0.9627	0.9765	3,202,419
YAC-Net	**97.8**	97.7	**0.9773**	**0.9913**	**1,924,302**

All the data in the table are the average of the prediction results of the test set after fivefold cross-validation of each model. The best results are shown in bold

In Table 2, all models have a precision and recall of more than 94%, and mAP_0.5 is not less than 0.96, which means that each model can accurately detect and classify the parasite egg images under the microscope. Further, it can be found that the YOLOv3 and YOLOv6n models have the most parameters, and the experimental metrics of the YOLOv6n model are higher than those of YOLOv3 in precision, recall, F1 score, and mAP_0.5. The experimental results may be due to the more advanced detection head and label allocation strategy of the YOLOv6n model. The number of parameters of the YOLOv8n has been significantly reduced compared with YOLOv3 and YOLOv6n. In terms of evaluation metrics, its detection performance is not inferior to YOLOv6n, and its recall is the highest among several methods. The parameters of YOLOv5n(Swin-T) are slightly higher than those of YOLOv8n, but the results in Table 2 present that its metrics are lower than those of YOLOv8n and YOLOv6n. Compared with YOLOv5n, the Swin-Transformer structure does not improve the model performance much, and its parameters are significantly higher than those of YOLOv5n. It can be concluded that Transformer is not suitable as the backbone of lightweight models. YOLOv5n has a weaker overall performance in data-based metrics, but its parameters are only higher than YAC-Net. Compared with several other detection methods, YOLOv5n achieves similar performance with the least parameters. This result highlights the excellent model structure of YOLOv5n. The YAC-Net designed by us has achieved the best performance in all metrics except recall, proving that the improvement of AFPN structure and C2f module based on YOLOv5n can effectively improve the detection ability of the model.

Figure 6 visualizes the results of six object detection methods for identifying parasite eggs in fecal sample images under a microscope. From the four parasite eggs shown in Fig. 6, we can see that parasite eggs have very different appearances under different shooting methods and different light intensities. Different parasite eggs have different sizes and orientations in the image, which is a huge challenge for the detection performance of the model. By analyzing the visualization results in Fig. 6, it can be concluded that for different eggs under different conditions, the six models can accurately locate the eggs and perform correct category judgment. Although the gaps in positioning among the models are difficult to reflect, there are certain differences in the confidence scores of egg classification by different methods. The egg in Fig. 6A is *Capillaria philippinensis*, which is smaller in size than other eggs, making them more difficult to identify. Among the six methods, YOLOv5n, YOLOv5n(Swin-T), and YOLOv6n performed slightly worse, while the recognition results of the other three methods were similar, with confidence scores all above 0.88. The egg in Fig. 6B is *Taenia* spp. egg, which are out-of-focus images. Analysis of the detection results shows that the models are all robust to out-of-focus images, and the classification confidence of the six methods is above 0.9, with only YOLOv3, YOLOv5n(Swin-T), and YOLOv8n below 0.96, and YAC-Net is only slightly lower than the best YOLOv6n model by 0.004 in confidence. Figure 6C shows *Ascaris lumbricoides* with noise, and the results of the models on this image are excellent, with YOLOv5n(Swin-T) and YOLOv8n’s confidence score slightly lower than 0.9, and YOLOv3 even achieved the highest score of 0.979. Figure 6D has both motion blur and low light conditions, which makes detection more difficult. Unexpectedly, all models correctly detect and classify the eggs, with YOLOv8 achieving the best detection score and YAC-Net ranking second with a confidence score of 0.936. Through the above analysis, it can be clearly seen that although YAC-Net has not achieved the best results in the above visualization results, all the eggs are correctly identified, and the gap with the best results is very small each time. Whether in the detection of small targets or in low-light motion blurred images, YAC-Net performs stably, which highlights its strong robustness. It has always maintained the second place in all confidence scores, which also shows that the model has balanced detection capabilities under various interference conditions, reflecting that the model has strong adaptability and generalization capabilities. Therefore, YAC-Net ensures strong detection capabilities while reducing resource loss, lowers the threshold for parasite egg detection in remote areas, and reduces dependence on relevant professionals.

## Discussion

In previous studies, various object detection networks in the RCNN and YOLO families have been used to detect parasites and eggs, and there are also many methods based on them that have been modified. Viet et al. [21] used Fast R-CNN to detect eight types of parasite eggs, and its mAP reached 0.9767. Wang et al. [33] selected the largest model structure YOLOv5x in the YOLOv5 series and cascade RCNN for egg detection and improved the backbone of cascade RCNN by combining Swin-Transformer. The improved cascade RCNN performance exceeded that of YOLOv5x. As a two-stage target detection algorithm proposed later, it is normal that the detection performance of the cascade RCNN algorithm is better than that of YOLOv5x under the same level. The advantage of the YOLO algorithm is that the detection process is implemented end-to-end and the detection speed is faster. Pun et al. [34] used the YOLOv2 to YOLOv7 models to detect root-knot nematodes. Their experimental results showed that among these detection models, YOLOv5 achieved the best performance. Compared with the two-stage detection algorithm, the one-stage algorithm is fast, easy to use, and has relatively low resource consumption [26]; compared with other YOLO models, the structure of YOLOv5 is relatively simple, with a higher degree of lightweighting and slightly fewer parameters. These characteristics are the prerequisites for our model selection, so we decided to use YOLOv5 as the basic model for our parasite egg detection research.

In addition, to explore the effect of the modification in each module during the lightweight process of YOLOv5n on the performance of the model in identifying parasite eggs, we performed ablation studies to show the performance results of parasite egg detection using YOLOv5n as the basic model, the basic model with AFPN and C2f modules separately, and the final version of YAC-Net. The experimental environment, experimental devices, parameter settings, and experimental data are consistent with those described in the “Dataset and experimental setup” section. The experimental results are presented in Table 3. All the data in the table are the average of the prediction results of the test set after a fivefold cross-validation of each model.

**Table 3:** Ablation studies

Model	AFPN	C2f	Precision (%)	Recall (%)	F1 score	mAP_0.5	Parameters
YOLOv5n	–	–	96.7	94.9	0.9578	0.9642	2,505,089
YOLOv5n	√	–	96.7	96.1	0.9640	0.9826	1,898,518
YOLOv5n	–	√	97.3	96.6	0.9697	0.9762	2,566,497
YAC-Net	√	√	97.8	97.7	0.9773	0.9913	1,924,302

The effect of different components on model detection performance

To explore whether AFPN is efficient in fusing feature information in the model, the neck of the YOLOv5n was rebuilt into an AFPN structure. From the results in Table 3, it can be found that the YOLOv5n with the AFPN performs better than YOLOv5n in other evaluation metrics, except for slightly lower precision than the original model. This can prove that the asymptotic structure of AFPN helps to promote the feature fusion of the neck part, making it easier for the model to learn feature information; the adaptive spatial fusion mechanism introduced in the structure allows the model to choose to learn feature information that helps to identify the results during the training process, forget redundant information, and make the model more focused on egg detection. Finally, the model with AFPN has a lower number of parameters but improves performance results, which also shows that the direction of lightweight model is correct.

To verify the effectiveness of the C2f module in extracting features from image semantic detail feature information and spatial context feature information, the C3 module in the backbone of the basic model was modified to the C2f module. The results in Table 3 show that compared with the YOLOv5n model, the performance of the model has been improved after being modified to the C2f module, indicating that the C2f module in the backbone can enhance the model’s ability to analyze images and improve the model’s feature extraction ability, which ultimately increases the model’s sensitivity to eggs and improves detection performance. The YAC-Net algorithm proposed in this experiment based on YOLOv5n and combined with AFPN and C2f is superior to previous algorithms in terms of precision, recall, F1 score, mAP_0.5, and parameters. The above results confirm the effectiveness of AFPN and C2f structures in improving the performance of parasite egg detection in the YOLOv5n model. Compared with YOLOv5n, the significant reduction in the number of parameters in YAC-Net also indicates that the lightweight results of the model are successful.

Due to the C2f module also exists in YOLOv8 and from the overall framework, YOLOv8 is similar to the YOLOv5 model, we decided to further explore the potential relationship between YAC-Net and YOLOv8. Therefore, we conducted comparative experiments on YOLOv5n, YOLOv8n, YOLOv8n with C3 module, referred to as YOLOv8n(C3), YAC-Net, and YAC-Net with C3 module, referred to as YAC-Net(C3). The experimental results are presented in Table 4. From Table 4, we can see that the gap between the detection results of YOLOv8n(C3) and YOLOv5n is very small, and the parameters are very close. Considering that their model structures are roughly the same, this result is reasonable. In addition, the results of each evaluation metric of YAC-Net(C3) are better than YOLOv8n(C3). There is no doubt that this is the credit of the AFPN module, which selects appropriate feature information for the model through adaptive feature selection, avoids the interference of redundant information, and greatly reduces the number of parameters. It can also be concluded from Table 4 that the performance improvement between YOLOv8n and YOLOv8n(C3) is greater than the performance improvement between YAC-Net and YAC-Net(C3). Obviously, this is at the expense of the number of parameters. To balance computational consumption and model performance, the C2f module used in YAC-Net sets all expansion factors in the module to 0.5, while the expansion factor of the BottleNeck block in the C2f module in YOLOv8n is 1. This is why the increase in parameters from YAC-Net (C3) to YAC-Net model is less than that from YOLOv8n (C3) to YOLOv8n. Of course, since the number of C2f modules used in YAC-Net is less than YOLOv8n, the increase of additional parameters is inevitable.

**Table 4:** Performance comparison of YOLOv8 and YAC-Net with C3 and C2f modules, respectively

Model	Precision (%)	Recall (%)	F1 score	mAP_0.5	Parameters
YOLOv5n	96.7	94.9	0.9578	0.9642	2,505,089
YOLOv8n	97.3	98.0	0.9754	0.9911	3,007,793
YOLOv8n(C3)	95.3	95.2	0.9520	0.9739	2,482,993
YAC-Net(C3)	96.7	96.1	0.9640	0.9826	1,898,518
YAC-Net	97.8	97.7	0.9773	0.9913	1,924,302

Finally, to prove that the performance improvement of our proposed YAC-Net in egg detection is statistically significant compared with the original YOLOv5n, we use a paired *t*-test with a confidence interval of 95% to compare the performance of the two groups of models. The paired *t*-test results of the two models’ evaluation metrics based on the fivefold cross-validation mode are presented in Tables 5 and 6. From the *P* value in Tables 5 and 6, it can be concluded that there is a significant difference in performance between YAC-Net and YOLOv5n. Therefore, compared with YOLOv5n, the YAC-Net designed by combining AFPN and C2f modules has statistically significant improvements in egg detection performance.

**Table 5:** Paired *t*-test results of precision and recall of YOLOv5n and YAC-Net in fivefold cross-validation mode

	Precision (%)				Recall (%)			
	YOLOv5n	YAC-Net	*t*	*P*	YOLOv5n	YAC-Net	*t*	*P*
Fold1	96.7	98.0	− 14.905	0.000118	94.5	97.4	−64.116	< 0.0001
Fold2	96.2	97.5			94.7	97.5		
Fold3	97.1	98.0			94.9	97.7		
Fold4	96.1	97.2			95.0	97.7		
Fold5	97.5	98.4			95.1	98.0

**Table 6:** Paired t-test results of F1 score and mAP_0.5 of YOLOv5n and YAC-Net in fivefold cross-validation mode

	F1 score				mAP_0.5			
	YOLOv5n	YAC-Net	*t*	*P*	YOLOv5n	YAC-Net	*t*	*P*
Fold1	0.9559	0.9766	− 45.709	< 0.0001	0.9602	0.9906	−13.631	0.000168
Fold2	0.9545	0.9747			0.9594	0.9911		
Fold3	0.9598	0.9782			0.9688	0.9911		
Fold4	0.9558	0.9746			0.9626	0.9909		
Fold5	0.9628	0.9822			0.9702	0.9926		

## Conclusions

This paper takes parasite eggs as the detection object and the microscope image of parasite eggs as the experimental data. Based on YOLOv5n, the C2f module is introduced into the backbone to enrich the gradient information, and by modifying the structure in the neck to AFPN, the hierarchical and asymptotic adaptive fusion mode of features is realized, which improves the detection performance of the model and successfully designs a lightweight detection model, YAC-Net. The experimental results show that compared with the basic model, YAC-Net improves the precision by 1.1%, the recall by 2.8%, the F1 score by 0.0195, the mAP_0.5 by 0.0271, and its parameters are reduced by one-fifth. This verifies that the cross-level feature information fusion of the AFPN and the rich use of gradient information characteristics of the C2f module can indeed ensure the detection capability of the model on the basis of lightweighting the model. Compared with some state‑of‑the‑art object detection models, our YAC-Net achieved the best results of 97.8%, 0.9773, 0.9913, and 1,924,302 in precision, F1 score, mAP_0.5, and parameters, respectively, proving that our model has excellent recognition ability and strong robustness for egg detection in the same lightweight detection model.

Despite this, our research still has shortcomings, mainly reflected in the fact that most of the eggs contained in the images in the dataset are single-target eggs, while there may be multiple targets in the microscope field of view in clinical testing. Therefore, our future work focuses on the following two aspects: (1) using experimental data training data of microscopic images containing multiple eggs to further improve the detection performance and the robustness of the model, and (2) under the condition of ensuring the existing detection results, design a more lightweight detection algorithm to further reduce the cost and make the automated detection of eggs more widely promoted.

## Data Availability

No datasets were generated or analyzed during the current study.
